# A Consistency Evaluation and Calibration Method for Piezoelectric Transmitters

**DOI:** 10.3390/s17050985

**Published:** 2017-04-28

**Authors:** Kai Zhang, Baohai Tan, Xianping Liu

**Affiliations:** 1 School of Geosciences and Technology, China University of Petroleum, Qingdao 266555, China; tanbaohai@upc.edu.cn; 2 Logging Branch of China National Petroleum Corporation Bohai Drilling Engineering CO. LTD., Tianjin 300280, China; 2005xinchen2006@163.com

**Keywords:** piezoelectric transducers, transmitter consistency, impedance analyzer, consistency evaluation, consistency calibration

## Abstract

Array transducer and transducer combination technologies are evolving rapidly. While adapting transmitter combination technologies, the parameter consistencies between each transmitter are extremely important because they can determine a combined effort directly. This study presents a consistency evaluation and calibration method for piezoelectric transmitters by using impedance analyzers. Firstly, electronic parameters of transmitters that can be measured by impedance analyzers are introduced. A variety of transmitter acoustic energies that are caused by these parameter differences are then analyzed and certified and, thereafter, transmitter consistency is evaluated. Lastly, based on the evaluations, consistency can be calibrated by changing the corresponding excitation voltage. Acoustic experiments show that this method accurately evaluates and calibrates transducer consistencies, and is easy to realize.

## 1. Introduction

Acoustic transducers are used to realize energy transformations between alternating electric signals and sonic signals, and these devices play an important role in many domains, such as industry [1], agriculture [2], military [3], and medicine [4]. These transducers can be divided into piezoelectric and magnetostrictive transducers according to their materials, and piezoelectric transducer technologies that use piezoelectric effect to detect energy transformation are known to be more mature and useful [2,5,6]. Transducers that transform electric energy into sonic energy are called transmitters, and those that transform sonic energy into electric energy are called receivers.

With the development of transducer-applied technologies, array transducer and transducer combination technologies are evolving rapidly [7,8]. For example, in the process of petroleum exploration, acoustic transmitters are well-ordered and phase-arrayed to control the orientation and enhance the energy of sound waves [9,10,11]; in the process of nondestructive testing, the inspection performances are limited to regular surfaces, and flexible phased-array techniques are used to compensate for surface irregularities and to fit the surface [12]. While adapting transmitter combination technologies, the parameter consistencies between each transmitter are extremely important because they can determine a combined effort directly. On the other hand, transmitter consistencies can influence the consistencies between each tool. Thereafter, transducers must be chosen to obtain good consistencies. However, transducer manufacturing technologies are very complex and have low production pass rates, and the consistencies are influenced by many factors and are difficult to guarantee. Thus, the consistencies between transmitters must be evaluated and calibrated.

To date, the reciprocity theorem is mostly used to measure transducer consistencies [13,14]. This method is accurate, but its experiment process is tedious and only suited for reciprocity transducers. Transducer electronic parameters can be measured by impedance analyzers and, in this study, a variety of transmitter acoustic energies caused by electronic parameter differences are analyzed, and a consistency evaluation and calibration method based on these parameters is proposed. Experiments show that this method can efficiently evaluate and calibrate transducer consistencies.

## 2. The Consistency Evaluation Method Based on an Impedance Analyzer

Impedance analyzers deliver low-level current into the object that needs to be measured, and the object’s resistance and reactant characteristics are then recorded. By changing the current frequency continuously, impedance analyzers accurately perform a wide range of frequency measurements [15]. Thereafter, many parameters and performances are obtained by calculating the resistances and reactant characteristics, such as the resonance frequency, half power points, anti-resonance frequency, maximum admittance, mechanical quality factor, free capacitance, static capacitance, dynamic capacitance, dynamic impedance, dynamic inductance, and electromechanical coupling factor.

Resonance is a phenomenon in which a vibrating system or external force drives another system to oscillate with greater amplitude at a specific preferential frequency. Frequencies at which the response amplitude is a relative maximum are known as the resonant (or resonance) frequencies of the system. At resonant frequencies, small periodic driving forces have the ability to produce large amplitude oscillations. The maximum conductance is the value at the resonance frequency, which is the reciprocal of dynamic resistance. The mechanical quality factor is a dimensionless parameter that describes the energy that a piezoelectric object consumes to overcome the internal friction at the resonant frequency, and it also characterizes an oscillator’s bandwidth relative to its center frequency. The piezoelectric object translates mechanical into electrical energy or electrical into mechanical energy, and the electromechanical coupling factor is used to describe the degree of these energy translations.

**Figure 1:**
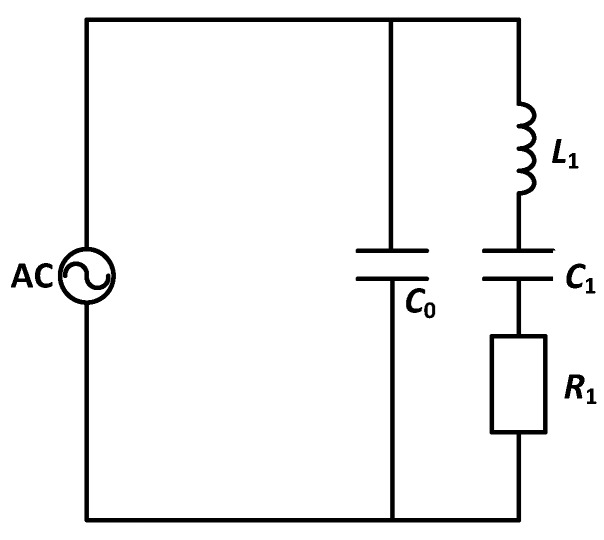
Equivalent circuit of a transmitter; only *C*_0_ is considered in static state, whereas *C*_1_, *R*_1_ and *L*_1_ must be considered connecting in series while vibrating.

A piezoelectric transducer can be regarded as only a capacitance (*C*_0_) in static state, whereas dynamic impedance must be considered while it is vibrating and emitting energy, which can be described by a capacitance (*C*_1_), a resistance (*R*_1_), and an inductance (*L*_1_) that are connected in series [16]. The equivalent circuit of the transducer is shown in Figure 1.

According to Ohm’s law, based on the assumption that the input voltage of the transducer is *U* at one moment, the instantaneous power of the equivalent circuit is as follows:

(1) $$P=UI=U^{2}(G_{f}+jB_{f})=U^{2}G_{f}+jU^{2}B_{f}$$

where *G_f_* and *B_f_* are the total conductance and the total susceptance at a certain frequency, *U*^2^*G_f_* is the real power and *U*^2^*B_f_* is the reactive power.

Based on the assumption that the spring constant in Hooke’s Law of one transducer is *k*, the average vibration displacement of the transducer in a very short time (Δ*t*) is Δ*X*, and according to Hooke’s law, the mechanical power is as follows:

(2) $$P_{0}=k\times \Delta X^{2}/\Delta t$$

Based on the assumption that all the electrical real powers are converted into mechanical power, then,

(3) $$U^{2}G_{f}=k\Delta X^{2}/\Delta t$$

Based on the aforementioned Equation (3), we conclude that the transducer conductance is proportional to the square of its displacement, whereas the input voltage (*U*), the spring constant (*k*), and the time (Δ*t*) are certain.

To confirm the aforementioned conclusion, analogue simulations were performed to the most common-used laminated transducer and cylindrical transducer by ANSYS software (Ansys Inc., Canonsburg, PA, USA) [17]. In the process of analogue simulations, PZT4 was used for piezo material, whose density is 7500 kg/m^3^. For the laminated transducer, the length of the piezoelectric ceramic block was 12 mm, while the width was 9 mm and the height was 1 mm. Meanwhile, the length of the substrate metal block was 24 mm, while the width was 9 mm and the height was 1 mm. The polarization of the piezoelectric ceramic block was along the through-thickness orientation. For the cylindrical transducer, the inner diameter for the piezoelectric ceramic block was 46 mm, the exterior diameter was 53 mm and the height was 48 mm. The polarization of the piezoelectric ceramic block was along the radial orientation. 

**Figure 2:**
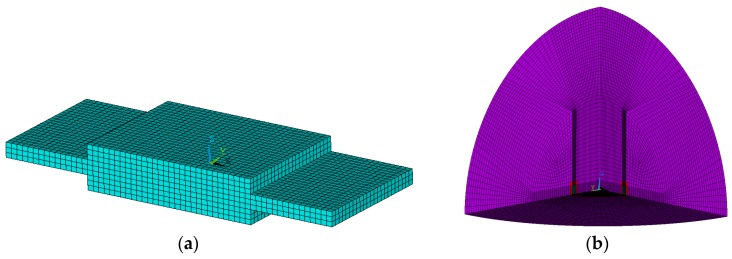

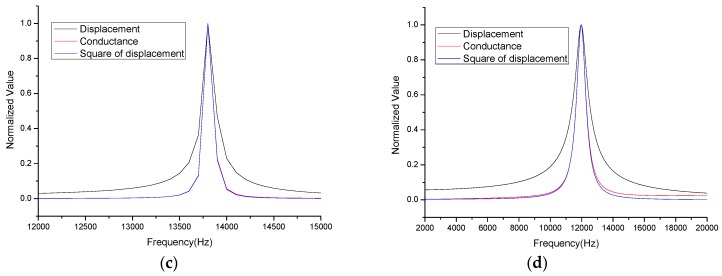
Finite element models for (**a**) laminated and (**b**) cylindrical transducers. Normalized simulation results ((**c**) for laminated and (**d**) for cylindrical transducers) indicate that the conductance curves coincide with the square of displacement curves.

Finite element models for laminated and cylindrical transducers are shown in Figure 2a,b. Normalized simulation results are presented in Figure 2c,d, where all conductance curves coincide with the square of displacement curves (especially at the frequencies near the resonance frequency). 

Transmitters should always work at nearby resonance frequencies. In the experiments and simulations, almost all the transmitter conductance curves are found to be similar to the Gaussian curve while working at those frequencies. Thereafter, the following empirical expression is proposed:

(4) $$G(F_{x},G_{max},Q_{m},F_{s})=G_{max}e^{\ln0.5\times 4Q_{m}{}^{2}/F_{s}{}^{2}\times {\left(F_{x}-F_{s}\right)}^{2}}$$

where *F_x_* is the working frequency, *F_s_* is the resonance frequency, *G_max_* is the maximum conductance, and *Q_m_* is the mechanical quality factor.

**Figure 3:**
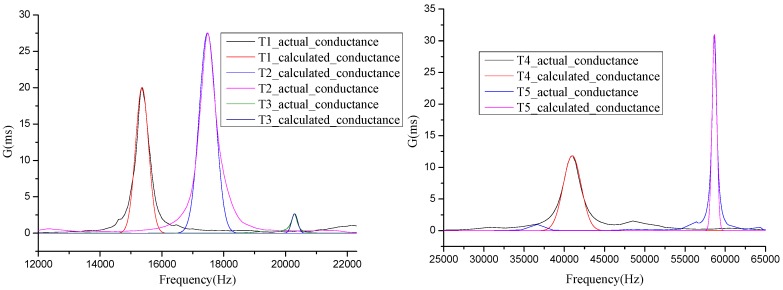
Calculated conductance curves based on the empirical expression and actual conductance curves measured by an impedance analyzer for five kinds of transducers: a laminated transducer (T1), a cylindrical transducer (T2), a sandwich lamination structure ultrasonic cleaning transducer (T3), an arc-shaped bending transducer (T4) and a much larger arc-shaped bending transducer isolated by rubber (T5).

Five transducers were selected and measured to confirm the aforementioned empirical expression, including one laminated transducer, one cylindrical transducer, one sandwich lamination structure ultrasonic cleaning transducer, one arc-shaped bending transducer and one much larger arc-shaped bending transducer isolated by rubber. Calculated conductance curves that are based on this empirical expression and actual measurement conductance curve are shown in Figure 3. Therefore, the calculated and actual values of nearby resonance frequencies are in substantial agreement.

When Equation (3) is substituted with Equation (4), the following equation can be obtained:

(5) $$\Delta X=\sqrt{\frac{U^{2}\Delta t}{k}G_{max}e^{\ln0.5\times 4Q_{m}{}^{2}/F_{s}{}^{2}\times {\left(F_{x}-F_{s}\right)}^{2}}}$$

As is well known, the sound pressure fired by one transmitter is proportional to the vibration displacement of its surface. Because the vibration displacement ΔX is difficult to measure, the peak-to-peak sound pressure value of the emitted acoustic signals is used to instead. Thereafter, transducers of the same material have the same spring constant (*k*), and their input voltage (U) and time (Δt) are determined values. The following equation is deduced:

(6) $$P_{\mathrm{sound}}\propto U\sqrt{G_{\max}e^{\ln0.5\times 4Q_{m}{}^{2}/F_{s}{}^{2}\times {\left(F_{x}-F_{s}\right)}^{2}}}$$

Equation (6) is deduced based on the assumption that all the electrical real powers are converted into mechanical power. However, part of the energy is consumed in the process of electromechanical coupling, and the electromechanical coupling factor (*K_P_*) is used to describe the degree of electromechanical coupling. On the other hand, piezoelectric transducers must consume energy to overcome internal friction, and the mechanical quality factor is used to describe it.

When the electromechanical coupling and mechanical quality factors are considered, Equation (6) can be described as follows:

(7) $$P_{\mathrm{sound}}\propto U\times K_{P}\sqrt{(1-2\pi /Q_{m})G_{max}e^{\ln0.5\times 4Q_{m}{}^{2}/F_{s}{}^{2}\times {\left(F_{x}-F_{s}\right)}^{2}}}$$

Equation (7) describes the relationship between sound pressures that are caused by working transmitters and the electronic parameters that are measured by impedance analyzers. That is, parameter consistencies of piezoelectric transmitters can be evaluated by measurement based on the impedance analyzers and the calculation based on Equation (7). As the calculation endings based on Equation (7) are proportionality values, the calculation results are normalized by dividing each result by the maximum result. After the influences of the electronic parameters have been calculated successfully, calibrations can be performed by adjusting the input voltages.

## 3. Experiments and Results

To validate the presented theory, eight laminated transmitters with resonant frequencies of about 15 kHz were chosen, and their electronic parameters were measured by an impedance analyzer. Thereafter, their normalized sound pressures were calculated by using Equation (7) (Table 1), where *K_P_*, *Q_m_*, *G_max_*, and *F_s_* denote electromechanical coupling factor, mechanical quality factor, maximum conductance, and resonance frequency, respectively.

**Figure 4:**
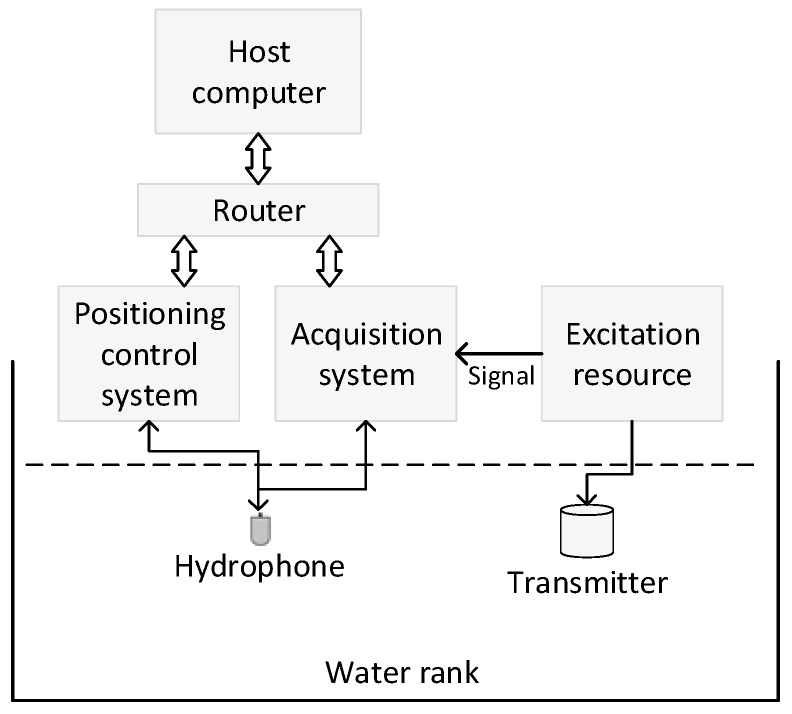
Experimental system for acoustic waveform measurements in a water tank with dimensions of 5.0 m × 5.0 m × 4.0 m. The positioning system allowed the precise adjustment of the relative position between the transmitter and the hydrophone. Positioning control system, acquisition system and host computer are connected by router and ethernet.

To measure their real sound pressure, the eight transmitters were emitted by the same electrical excitation source with a frequency of 15.5 kHz and an amplitude of 600 V, and the acoustic signals were received by the broadband hydrophone. The acoustic experiments were conducted in a water tank with the dimensions of 5.0 m × 5.0 m × 4.0 m. The fluid density and velocity were 1000 kg/m^3^ and 1500 m/s, respectively. As shown in Figure 4, the experimental apparatus included an electrical excitation resource, cylindrical transmitter, host computer, positioning control system, multichannel acquisition system, gain-controlled amplifier, and hydrophone [18,19]. The excitation resource produced high-voltage pulses to emit the transducer and synchronize signals to start the high-accuracy synchronous data acquisition system. The hydrophone and transmitters were accurately positioned with the positioning control system.

**Figure 5:**
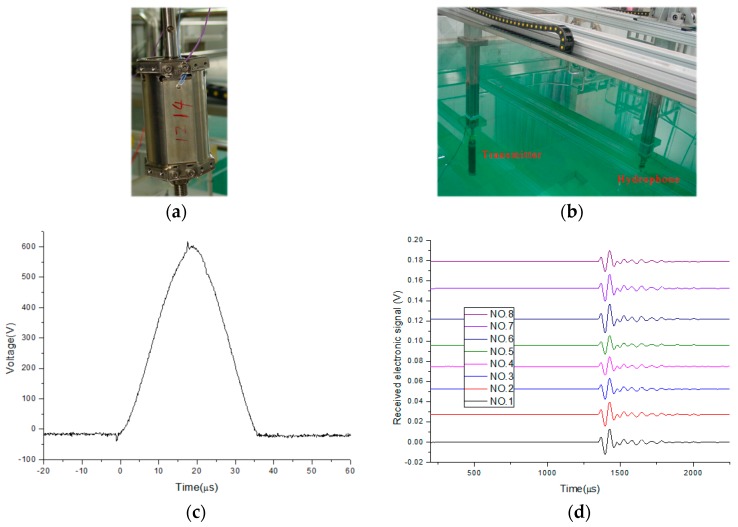
The diagram of one laminated transmitter (**a**), the diagram of the positioning control system (**b**), the firing voltage curve excited by the excitation resource (**c**), and eight sound pressure curves collected by the hydrophone (**d**) while eight transmitters were being excited, respectively.

**Figure 6:**
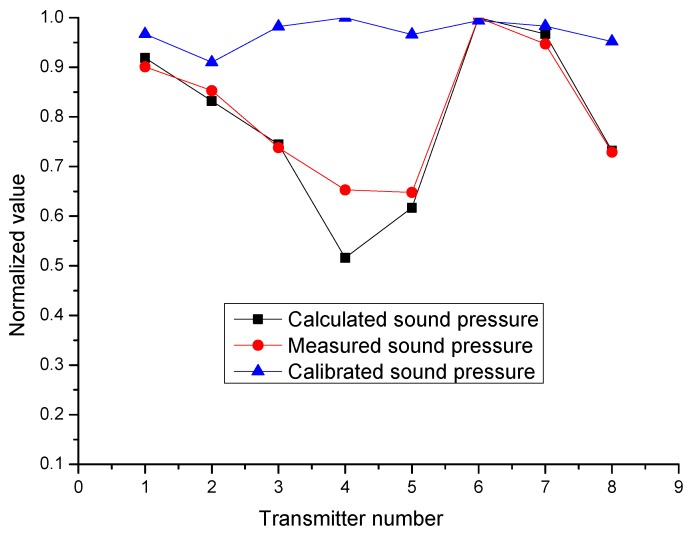
The calculated sound pressure curve of eight laminated transmitters based on Equation (7) (see Table 1), the measured sound pressure curve obtained by the measurement system in a water tank, and the calibrated sound pressure curve measured by the same system after excitation voltages for each transmitter were adjusted according to Equation (7), respectively.

The diagram of a laminated transmitter and the positioning control system are shown in Figure 5. The distance between the hydrophone and laminated transmitters was two meters. Eight transmitters were fixed in one octagonal mounting bracket, and the angle between neighboring transmitters was 45 degrees. In the process of the experiments, only one of these eight transmitters was opposite the hydrophone. While the selected transmitter was being excited by the excitation resource, the hydrophone collected the sound pressure curves. Then, the mounting bracket rotated 45 degrees, causing another transmitter to become excited, and the sound pressure curves were subsequently collected. The firing voltage curve and eight sound pressure curves are shown in Figure 5. Lastly, the normalized calculated and measured sound pressure are shown in Figure 6 and Table 2.

As different transducers were excited by different excitation resources, the excitation voltages for each transmitter could be adjusted by the logging tools to make up for the lack of consistency. The adjustment of input excitation voltages can be calculated according to Equation (7). Then, the acoustic experiments were repeated while each transducer was excited by its own excitation voltage. The adjustment of excitation voltages and calibrated sound pressures are shown in Table 2 and Figure 6. 

## 4. Discussion and Conclusions

The calculated and measured results show that the consistencies between transducers are not satisfactory. Table 2 and Figure 6 show that the calculated results are in accord with the experimental measurement results. Thereafter, by adjusting the excitation voltages for each transmitter, the consistencies between transducers were greatly improved. There are some discrepancies in the measured pressures after the voltage adjustment, because the acoustic experiment method had many influencing factors making the endings uncertain, such as the fixed strength of the transducer, the acoustic coupling condition, and the gas and oil bubbles outside of the rubber sheath, which change with time. Also, as the excitation voltage was adjusted by hand (a voltage regulator), it was not perfectly accurate, either.

This study introduces the electronic parameters of piezoelectric transmitters that can be measured by impedance analyzers. Thereafter, the relationship between these parameters and the transmitter acoustic performances is analyzed, and a consistency evaluation and calibration method for these transmitters is proposed. Acoustic experiments show that this method can evaluate and calibrate transducer consistencies. Compared with the traditional reciprocity theorem, this method is easier to realize and is suited for all transducers (not only reciprocity transducers).

## Figures and Tables

**Table 1:** Electronic parameters of the eight laminated transmitters and their normalized sound pressure calculation results based on Equation (7). *K_P_*, *Q_m_*, *G_max_*, and *F_s_* denote electromechanical coupling factor, mechanical quality factor, maximum conductance, and resonance frequency, respectively.

NO.	*K_P_*	*Q_m_*	*G_max_* (ms)	*F_S_* (Hz)	Normalized Calculation Results
1	0.432	36.941	21.242	15368	0.919
2	0.428	40.474	23.018	15496	0.832
3	0.431	32.116	17.715	15420	0.745
4	0.401	17.817	10.727	15268	0.516
5	0.400	29.354	15.584	15500	0.617
6	0.380	39.903	22.739	15686	1.000
7	0.371	27.454	17.331	15840	0.967
8	0.399	34.420	20.204	15560	0.732

**Table 2:** The calculated sound pressures of eight laminated transmitters based on Equation (7); the measured and normalized peak-to-peak sound pressures measured by the acoustic waveform measurement system; the adjustment excitation voltages for each transmitter depending on the measured sound pressures and Equation (7); the measured peak-to-peak (*) and normalized sound pressures (*) measured by the same system after voltage adjustment.

NO.	Calculation Results	Measured Sound Pressure (Pa)	Normalized Pressure	Adjustment Voltage (V)	Measured Sound Pressure * (Pa)	Normalized Pressure *
1	0.919	1386.4	0.901	665.9	1539.9	0.967
2	0.832	1312.6	0.853	703.4	1449.1	0.910
3	0.745	1135.6	0.738	813.0	1563.8	0.982
4	0.516	1004.8	0.653	918.8	1592.5	1.000
5	0.617	997.1	0.648	925.9	1538.3	0.966
6	1.000	1538.8	1.000	600	1582.9	0.994
7	0.967	1457.2	0.947	633.6	1565.4	0.983
